# Neural correlates of emotional processing in psychosis risk and onset – A systematic review and meta-analysis of fMRI studies

**DOI:** 10.1016/j.neubiorev.2021.03.010

**Published:** 2021-09

**Authors:** P.B. Lukow, A. Kiemes, M.J. Kempton, F.E. Turkheimer, P. McGuire, G. Modinos

**Affiliations:** a Department of Psychosis Studies, Institute of Psychiatry, Psychology & Neuroscience, King’s College London, De Crespigny Park, SE5 8AF, London, UK; b Department of Neuroimaging, Institute of Psychiatry, Psychology & Neuroscience, King’s College London, De Crespigny Park, SE5 8AF, London, UK; c MRC Centre for Neurodevelopmental Disorders, King’s College London, new hunt’s House, Guy’s Campus, SE1 1UL, London, UK

**Keywords:** Emotion, Psychosis, Clinical high-risk, Functional magnetic resonance imaging, Seed-based d mapping

## Abstract

Aberrant emotion processing is a well-established component of psychotic disorders and is already present at the first episode of psychosis (FEP). However, the role of emotion processing abnormalities in the emergence of psychosis and the underlying neurobiology remain unclear. Here, we systematically reviewed functional magnetic resonance studies that used emotion processing task paradigms in FEP patients, and in people at clinical high-risk for psychosis (CHRp). Image-based meta-analyses with Seed-based *d* Mapping on available studies (n = 6) were also performed. Compared to controls, FEP patients showed decreased neural responses to emotion, particularly in the amygdala and anterior cingulate cortex. There were no significant differences between CHRp subjects and controls, but a high degree of heterogeneity was identified across studies. The role of altered emotion processing in the early phase of psychosis may be clarified through more homogenous experimental designs, particularly in the CHRp population.

## Highlights

• The neural bases of altered emotion processing in psychosis are still unclear.
• Systematic review indicated widespread activation decreases to emotion in first-episode psychosis.
• Evidence in people at clinical high-risk for psychosis lacked convergence.
• These findings were corroborated by image-based meta-analyses.

## 1 Introduction

Emotional abnormalities, including alexithymia and blunt affect, are an established component of the presentation of psychotic disorders such as schizophrenia (van Os and Kapur, 2009; Kahn et al., 2015). There is consistent evidence for aversion to positive and neutral stimuli in schizophrenia (Cohen and Minor, 2010) and facial emotion recognition deficits (Chan et al., 2010; Kohler et al., 2010), which could be valence- or task difficulty-dependent (Johnston et al., 2001). However, the neurobiology underlying the development of such abnormalities and their putative role in psychosis development remain unknown. Several meta-analyses of functional magnetic resonance (fMRI) and positron emission tomography (PET) studies showed lower activation to emotional stimuli compared to neutral stimuli in schizophrenia patients relative to healthy controls in several brain regions (Kohler et al., 2010; Anticevic et al., 2012; Taylor et al., 2012; Li et al., 2010). Such finding was most consistently reported for the bilateral amygdala in relation to aversive stimuli (Anticevic et al., 2012) as well as to facial emotion expressions (across valences, analysing threatening expressions only, or isolating implicit and explicit processing) (Taylor et al., 2012; Li et al., 2010; Dong et al., 2018). Under-recruitment of other regions to emotion-related stimuli was also reported, such as in the right superior frontal gyrus (Li et al., 2010), hippocampus, early visual processing regions, frontal cortices (Taylor et al., 2012) and fusiform gyrus (Dong et al., 2018). These meta-analyses also identified aspects of study design that may underlie region-specific differences across studies; for instance, implicit or explicit task paradigms may recruit disparate brain networks (Li et al., 2010) and comparison to a neutral condition may yield more robust results than a comparison across emotion conditions (Anticevic et al., 2012). With evidence showing that antipsychotic medication does not adequately treat emotion-related deficits in patients with schizophrenia (Kohler et al., 2010) and that emotion processing deficits in schizophrenia are predictive of functional outcome (Kee et al., 2003), studying populations in the early stages of psychosis may inform research into more targeted therapies as well as potential new preventative treatments (Davies et al., 2018).

Despite an increase in the literature on the neural correlates of emotion processing in first episode of psychosis (FEP) and at-risk populations in the last decade, no systematic review and meta-analysis to date has attempted to synthesise this evidence. While an impairment in facial emotion recognition across positive and negative valences is reported as already present at the first episode (Barkl et al., 2014; Bosnjak Kuharic et al., 2019), it is unclear whether this is also the case in people at clinical high risk for psychosis (CHRp) (Fusar-Poli et al., 2013). Individuals at CHRp present with subtle, subjective disturbances in attention and cognition (Ruhrmann et al., 2010; Klosterkötter et al., 2001), attenuated psychosis symptoms and functional decline (Fusar-Poli et al., 2013; Keshavan et al., 2011; Fusar-Poli et al., 2015a). Emotion processing was reported to be impaired across at-risk, FEP and chronic schizophrenia samples (Green et al., 2012), suggesting that it may be a trait characteristic. However, other behavioural studies in CHRp showed contrasting results. Some found no deficit in emotion recognition (Thompson et al., 2012; Addington et al., 2012), while others reported impaired neutral face recognition (Van Rijn et al., 2011) and poorer sad and fearful face recognition (Amminger et al., 2012). A putative explanation for such disparate results is that they might be dependent on the future transition status of CHRp participants, as it was shown that poorer neutral, and better fearful expression recognition was associated with transition status (Allott et al., 2014), and more recently, that poor functional outcome is associated with anger recognition in individuals at CHRp (Modinos et al., 2020). Furthermore, behavioural manifestation of emotion processing abnormalities can be preceded by the development of underlying biological mechanisms (Keshavan et al., 2011). In their recent systematic review on neural correlates of social cognition (processing of socially-relevant stimuli) in psychosis proneness, Kozhuharova and colleagues found convergent activation increases in the lateral temporal cortex to emotional and neutral stimuli in CHRp individuals compared to controls, but inconsistent results for the frontal cortex and limbic regions (Kozhuharova et al., 2019). The only meta-analysis of fMRI studies on emotion processing in FEP to date, using a coordinate-based approach (activation likelihood estimation), did not find differences in brain activation to emotional stimuli compared to healthy controls, although within-group analyses suggested that the FEP group recruited fewer regions (Del Casale et al., 2018). The aim of our study was to conduct an up-to-date systematic review of fMRI studies on emotion processing in the FEP and CHRp states, complemented by a robust image-based meta-analysis from available studies, which is considered a more sensitive approach than coordinate-based methods (Radua et al., 2012). We hypothesised a clear pattern of altered activation in FEP compared to healthy controls during emotional processing that would be less pronounced in those at CHRp.

The present study aimed to systematically review and meta-analyse fMRI studies investigating emotion processing in FEP and CHRp. We focused on discrete, non-compound and culturally universal emotions (anger, fear, happiness, sadness, disgust or surprise) (Ekman and Cordaro, 2011)). We performed the first systematic review on this topic within the FEP population and expanded on that by Kozhuharova et al. within the CHRp population, as we discern immediate emotion recognition or discrimination from cognitively demanding tasks regardless of stimulus type. We took a critical approach and appraised the putative influence of task paradigm and participant inclusion criteria when considering the findings. Where applicable, we also appraised the brain response to the neutral condition, which is often used as a comparator in emotion processing studies. We then meta-analysed the available unthresholded statistical images of group comparisons from reviewed studies with Seed-based *d* Mapping (SDM), a method which has been shown to have higher sensitivity than coordinate-based meta-analyses (Radua et al., 2012). This way, we reviewed the neural correlates of emotion processing in FEP and CHRp and performed a meta-analysis of CHRp studies for the first time. Finally, we discussed the findings in light of current hypotheses on the role of emotion processing in the development of schizophrenia.

## 2 Methods

### 2.1 Study selection for the systematic review and meta-analysis

The MEDLINE database was searched via PubMed and Ovid interfaces for published functional neuroimaging articles in either people at CHRp or with an FEP compared to a healthy control sample, during an emotion processing task, until 03 July 2019. The search terms included ‘high risk’, ‘first’, ‘episode’, ‘psychosis’, ‘function*’, ‘emotion*’ (for the full list, see Supplement). Initially, fMRI, arterial spin labelling (ASL), single photon emission computed tomography (SPECT) and PET studies were searched for. The inclusion criteria were selected to maximise study homogeneity and consisted of: an emotion processing paradigm (i.e., the recognition or discrimination of discrete, non-compound and culturally universal emotions (Ekman and Cordaro, 2011), but not social or cognitive tasks such as theory of mind, working memory or reappraisal), task performance during a functional neuroimaging scan and a validated form of clinical assessment for inclusion of participants through a structured interview. The inclusion criteria for the FEP group comprised short duration of illness (maximum of two years) and a diagnosis of schizophrenia, schizophreniform disorder, brief psychotic disorder or affective psychosis according to the Diagnostic and Statistical Manual of Mental Disorders, version IV or 5 (DSM-IV or DSM-5) or International Classification of Disorders, version 10 (ICD-10) F20−29 or F31−33 (American Psychiatric Association, 2013; WHO, 2016). In case of a very short duration of illness, i.e. below the 6-month clinical period required by DSM for schizophrenia diagnosis, severe symptomatology assessed by the Positive and Negative Syndrome Scale (PANSS) (Kay et al., 1987) warranting inpatient admission was also considered (Villalta-Gil et al., 2013). For CHRp, Comprehensive Assessment of At Risk Mental States (CAARMS) or Structured Interview for Prodromal Syndromes and the Scale of Prodromal Symptoms (SIPS/SOPS) were permitted (Yung et al., 2005; Miller et al., 2003), but not self-report questionnaires (used for the assessment of schizotypy, a separate at-risk group (Linscott and Van Os, 2013)) or a purely genetic/familial risk paradigm to focus on clinical risk for psychosis. Quality of studies was assessed alongside data extraction on a pre-generated standardised data extraction form including items necessary for study replication, in line with the current recommendations for fMRI studies (Nichols et al., 2017). The extracted data involved sample inclusion criteria, task paradigm and stimuli used, details of acquisition parameters including scanner type, statistical method and software used, outlier handling and consistency in result reporting. Both quality assessment and outcome measure extraction from studies was performed by two independent researchers (PBL and AK) and any disparities were clarified through discussion until a consensus was reached.

### 2.2 Systematic review - outcome measures

The primary outcome measure extracted from each study was the result of a comparison between brain activation in either FEP or CHRp relative to healthy controls, during the performance of an emotion processing task. Region where an effect was found and the *p* value reported from either a whole-brain or a region of interest (ROI) analysis were extracted. Only whole-brain images were used for the meta-analysis. Further details can be found in the Supplement regarding study heterogeneity, in terms of recorded task paradigm, stimuli type (facial/non-facial; valence), analysis contrast, between-group comparison statistical method and medication status of participants (see Supplement). For the systematic review, all reported results were recorded to avoid selection bias. Results for the emotional condition were then grouped by brain region and are reported below when convergent in at least two articles. Subsequently, to synthesise all existing evidence on the response to the neutral condition in FEP and CHRp, all relevant results were recorded and reported regardless of convergence.

### 2.3 Meta-analysis

The studies identified for the systematic review were then assessed for eligibility for meta-analysis. The choice of studies included in the quantitative meta-analysis was based on the similarity of tasks used to maximise the homogeneity of methodologies, according to the current standards for neuroimaging meta-analyses (Müller et al., 2018). One study was excluded from meta-analysis on this basis, as it concerned an aversive conditioning paradigm (Quarmley et al., 2019). Since there is limited evidence on valence-specific regional brain activation (Murphy et al., 2003; Lindquist et al., 2012), task paradigm involving immediate recognition of emotion was prioritised over valence homogeneity, as was done in previous neuroimaging meta-analyses of emotion in schizophrenia (Taylor et al., 2012; Li et al., 2010). Whole-brain, unthresholded group comparison images (t-maps) were used for the meta-analysis, to maximise sensitivity (Albajes-Eizagirre et al., 2019). Accordingly, ROI group comparison t-maps were excluded. To include images only normalised to the same standardised space, the more common MNI template was prioritised. Study authors were contacted directly to access raw images of the individual studies. The images requested were group comparison unthresholded t-statistic maps (t-maps) between a clinical population (FEP / CHRp) and a healthy control sample. SDM 6.21 meta-analyses raw study t-map images by transforming them into Hedge’s *d* effect-size maps, inferring individual subject images through multiple imputation, and meta-analysing the images through fitting a random-effects model (Radua et al., 2012). The default recommended parameters during pre-processing (a grey matter mask and a 20 mm anisotropic FWHM kernel), and 1,000 imputations for the FWE correction were used. An uncorrected threshold of p = 0.005 was used for the initial search, to then determine significant effects at a threshold-free cluster enhancement threshold (TFCE) of p_TFCE_< 0.05. TFCE was used, as it was validated as neither too conservative nor too liberal in neuroimaging meta-analysis performed with SDM (Albajes-Eizagirre et al., 2019). In brief, TFCE enhances sensitivity of the analysis by incorporating both peak height as well as cluster extent in result calculation, thus avoiding pre-defined arbitrary cluster extent threshold (Smith and Nichols, 2009). Where in a single study the same participants performed more than one relevant task, the images were combined using the Combine images tool in SDM to include only original samples in the final meta-analysis. The same process was performed for studies which shared their samples (Modinos et al., 2015; Tseng et al., 2016). Heterogeneity statistics were extracted for peaks returned by meta-analysis directly from I^2^, Q^2^ and H^2^ maps generated by SDM and displayed in MRIcron v1.0.20190902. These are reported in the Supplement in Table S2 as well as Figure S1 (including a whole-brain image of the I^2^ statistic distribution, recommended by the SDM developers to indicate the degree of variation in result estimate attributable to study heterogeneity (Higgins and Thompson, 2002).

## 3 Results

### 3.1 Studies included in systematic review and meta-analysis

**Fig. 1:**
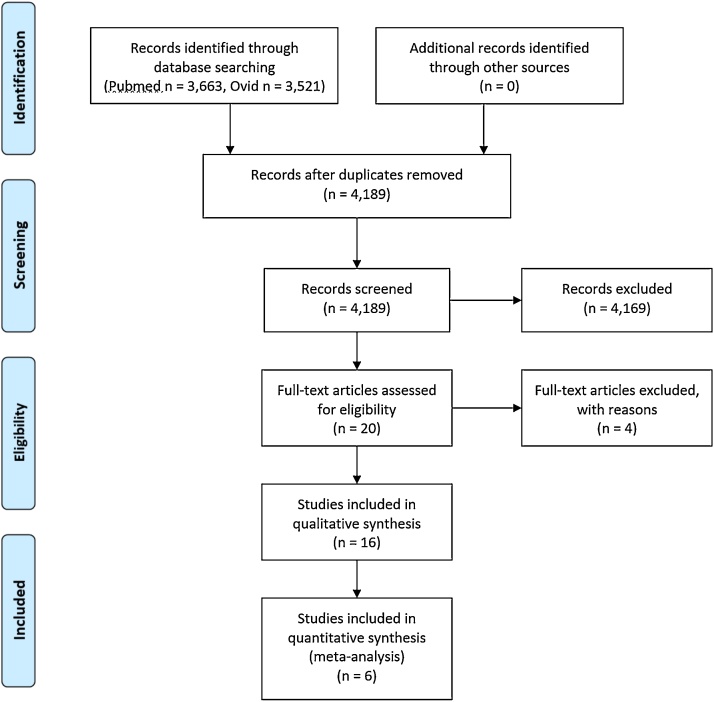
PRISMA diagram of the systematic review search process.

The PRISMA diagram of the systematic review search process (Moher et al., 2009) is depicted in Fig. 1 (see Supplement for the full PRISMA checklist). A total of 4,189 non-duplicate records were retrieved during the search. After initial screening, 19 potentially relevant fMRI studies and 1 PET study were identified. Further, full-text assessment of candidate eligibility was performed, followed by reference screening of the identified articles. Three fMRI studies were excluded due to 1) inappropriate statistical analysis (i.e., no group comparison), 2) at-risk state being identified via self-report and not via a structured clinical interview, and 3) inconsistency between statistical analysis and reported results (i.e., unmet quality assessment criteria). The PET study was excluded as the emotion processing task was applied before the scan and association between the two was not analysed. No SPECT or ASL studies directly investigating associations with emotion processing were found.

Sixteen fMRI papers were included in the systematic review, of which five assessed individuals at CHRp, nine included FEP patients, and two comprised both CHRp and FEP samples. Of these sixteen papers, twelve were found eligible for meta-analysis. Of the twelve eligible studies, six were made available by the authors, including the two which assessed both an FEP and a CHRp sample compared to the same group of healthy controls. Table 1 provides details of the studies included in the systematic review and reasons for exclusion from meta-analysis. Table S1 provides methodological details of studies included in the meta-analysis.

**Table 1:** Main methodological details of studies included in the systematic review and meta-analysis (green).

CHRp, clinical risk for psychosis; FEP, first episode of psychosis; IAPS, International Affective Picture System.

### 3.2 Systematic review – results in FEP patients

The most commonly reported finding in FEP patients was decreased activation of the amygdala in response to emotional stimuli, in six of eleven studies (Modinos et al., 2015; Tseng et al., 2016; Yang and Wang, 2017; Bergé et al., 2014; Das et al., 2007; Knolle et al., 2018). Attenuated responses were also common in the anterior cingulate (ACC) (Das et al., 2007; Knolle et al., 2018; Hempel et al., 2003; Reske et al., 2007), medial frontal / prefrontal (Bergé et al., 2014; Das et al., 2007; Hempel et al., 2003; Reske et al., 2009; Catalucci et al., 2011; Ebisch et al., 2013), and lingual cortex (Tseng et al., 2016; Yang and Wang, 2017; Bergé et al., 2014; Knolle et al., 2018), with four studies reporting findings in each of these regions. Attenuated responses were also reported in the thalamus (Yang and Wang, 2017; Bergé et al., 2014; Das et al., 2007), hippocampus (Bergé et al., 2014; Knolle et al., 2018; Hempel et al., 2003), inferior frontal gyrus (Reske et al., 2009; Catalucci et al., 2011), left postcentral gyrus (Bergé et al., 2014; Reske et al., 2009), frontal operculum (Bergé et al., 2014; Knolle et al., 2018), angular gyrus (Reske et al., 2009; Ebisch et al., 2013) and cerebellum (Bergé et al., 2014; Knolle et al., 2018).

These studies used a diversity of task paradigms, stimuli types, analysis contrast and stimuli (uniformity of task paradigms and sample characteristics can be found in the Supplement). No lateralisation of results was evident. Overall, attenuated responses to emotional stimuli in FEP subjects relative to controls were more common than increases in activation. The most consistent findings of activation increases were in the posterior cingulate cortex (PCC) (Hempel et al., 2003; Reske et al., 2009) and precuneus (Reske et al., 2009; Catalucci et al., 2011). Two studies reported no significant group differences with facial emotion tasks (Villalta-Gil et al., 2013; Tseng et al., 2016).

Only two studies reported results for a neutral condition. Modinos et al. compared a neutral condition to a baseline condition (fixation cross) and found greater activation in the left inferior frontal gyrus/anterior insula, as well as in bilateral amygdala (Modinos et al., 2015) in FEP relative to controls. Reske et al. reported attenuated activation of the left orbitofrontal region to the neutral condition in FEP subjects, but greater activation to neutral than to sad or happy faces in the hippocampus (Reske et al., 2009).

### 3.3 Meta-analysis – results in FEP patients

Five t-maps from four studies were available for meta-analysis (Table S1) (Modinos et al., 2015; Tseng et al., 2016; Bergé et al., 2014; Knolle et al., 2018). Tseng et al. and Modinos et al. largely shared their participants, hence the t-maps from their studies were combined using the ‘Combine images’ tool in SDM, and the degrees of freedom were calculated for the average sample size (as in Norman et al., 2016). Therefore, the final meta-analysis pooled data from a total of 48 patients and 73 healthy controls.

**Fig. 2:**
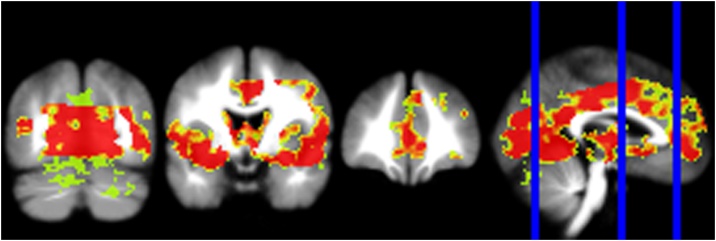
Results of the meta-analysis of group-comparison t-maps in the FEP population compared to healthy controls. Results shown on a standard template at p_TFCE_<0.001 for display purposes. Decreasing Z value displayed with increasing warm colours (green, yellow, red).

The meta-analysis of t-maps of brain activation to emotional versus neutral stimuli in FEP patients compared to healthy controls indicated that patients showed significantly decreased activation in a large widespread cluster (Z = -(5.284−1.631), k = 63,496, p_TFCE_ < 0.05). The cluster comprises peaks in several brain regions classically involved in emotion processing, such as the left insula (x = -38, y = 2, z = -10, Z = -4.655, p_TFCE_ = 0.000999987), left amygdala (x = -26, y = -2, z = -14, Z = -4.027, p_TFCE_ = 0.000999987), right hippocampus (x = 28, y = -38, z = 4, Z = -3.950, p_TFCE_ = 0.000999987), left hippocampus (x = -18, y = -10, z = -12, Z = -4.093, p_TFCE_ = 0.000999987), anterior cingulate (x = 0, y = 40, z = 4, Z = -3.611, p_TFCE_ = 0.000999987), and occipital cortex (x = -42, y = -72, z = 14, Z = -3.565, p_TFCE_ = 0.000999987) (Fig. 2). See Supplement for full cluster information (Table S2) as well as heterogeneity analyses (Table S3, Figure S1).

### 3.4 Systematic review – results in people at CHRp

Two articles of the seven suitable for the review reported greater activation of the PCC in CHRp than in healthy controls (Derntl et al., 2015; Van Der Velde et al., 2015). Four studies reported differential responses in the inferior frontal gyrus/ventrolateral prefrontal cortex: two found less activation in the CHRp group (Modinos et al., 2015; Seiferth et al., 2008), another found less activation, but only after combining the CHRp and schizophrenia groups (Quarmley et al., 2019), and one found a greater response (Derntl et al., 2015). No other results were convergent between studies. Two studies found no significant group differences (Quarmley et al., 2019; Tseng et al., 2016).

Three studies assessed the fMRI response to neutral stimuli. Seiferth et al. compared the activation parameters from regions that showed an interaction between the facial emotion recognition task and group (CHRp and HC), i.e., from inferior frontal gyri for happy and neutral; left superior frontal gyrus for angry, neutral and fearful; and left thalamus for sad and neutral stimuli. They found greater activations to neutral stimuli than the respective emotional conditions in the CHRp group compared to healthy volunteers in all these regions. Comparing the neutral condition to a fixation cross baseline, Modinos et al. found greater activations in left inferior frontal gyrus/anterior insula in the CHRp group compared to controls. Finally, van der Velde et al. compared the neutral condition to a blank screen baseline and found less activation in left temporal pole and bilateral PCC in CHRp.

### 3.5 Meta-analysis – results in people at CHRp

For the CHRp meta-analysis, six t-maps from four studies were collected (Table S1) (Modinos et al., 2015; Tseng et al., 2016; Van Der Velde et al., 2015; Seiferth et al., 2008). The maps from the studies by Tseng et al. and Modinos et al. were combined as for the FEP meta-analysis. The same process was performed for the two maps provided from van der ​Velde et al. from group comparisons of activation to different attentional dimensions (‘View’ and ‘Attend’) of the same emotion > neutral comparison on the same sample, relevant to the scope of this meta-analysis (Table S1). Therefore, the final meta-analysis pooled data from a total of 45 CHR individuals and 48 healthy controls.

**Fig. 3:**
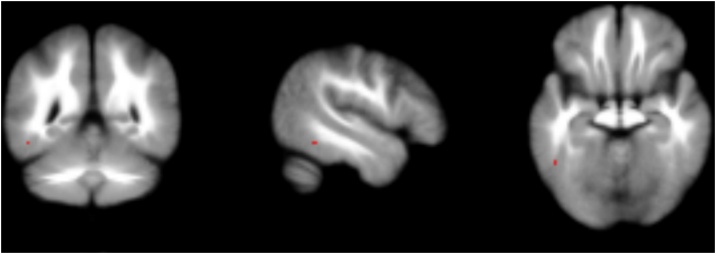
Results of the meta-analysis of group-comparison t-maps in the CHRp population compared to healthy controls. Results shown on a standard template at p_uncorr_<0.005 for display purposes.

The meta-analysis of t-maps of brain activation to emotional versus neutral stimuli in individuals at CHRp compared to healthy controls returned one small cluster of decreased activation to emotional stimuli in the left inferior temporal gyrus at an uncorrected level (x = -48, y = -50, z = -14, Z = -2.719, k = 3, p_uncorr_ = 0.003) (Fig. 3). This result did not survive p_TFCE_ < 0.05 correction. Since this meta-analysis returned no corrected effects, a heterogeneity analysis was not performed.

## 4 Discussion

The main finding of our systematic review with meta-analysis is that patients with an FEP display attenuated neural responses to emotional stimuli relative to neutral stimuli, compared to that of healthy controls, in brain regions implicated in emotion processing (referred to as hypoactivation below), such as the amygdala and ACC. In individuals at CHRp, relative convergence between studies involved greater neural responses in the PCC relative to healthy controls. However, this was only reported in two of seven studies, and was not evident in meta-analysis.

The finding of hypoactivation of the amygdala in FEP patients is consistent with previous work in chronic schizophrenia (Anticevic et al., 2012; Taylor et al., 2012; Li et al., 2010; Dong et al., 2018) and supports previous suggestions that altered amygdala response to emotion is stable across disease stage (Aleman and Kahn, 2005). However, a previous coordinate-based meta-analysis found no difference between FEP patients and healthy controls in amygdala recruitment during emotion processing, although within-group analyses suggested a more extensively recruited network in healthy controls than in FEP patients (Del Casale et al., 2018). This inconsistency may stem from a more sensitive methodology being used in the present study through an image-based meta-analysis, which is found to be more sensitive to coordinate-based methods (Radua et al., 2012). In the healthy brain, amygdala recruitment is a robust finding in neuroimaging studies of emotion (Murphy et al., 2003; Lindquist et al., 2012; Vytal and Hamann, 2010; Kirby and Robinson, 2017; Sabatinelli et al., 2011). It has been proposed that the role of the amygdala relates to determining the emotional salience of a stimulus, independently from its affective appraisal (Lindquist et al., 2012; Aleman and Kahn, 2005; Blackford et al., 2010; Phillips et al., 2003). Hence, amygdala hypoactivation in psychosis has been hypothesised to result from either poor recruitment in the processing of emotional stimuli (Li et al., 2010); increased tonic responsivity to non-salient, neutral stimuli (Anticevic et al., 2012; Taylor et al., 2012; Dong et al., 2018; Dugré et al., 2019); or both (Aleman and Kahn, 2005). On an individual level, the result of an fMRI contrast depends on both the response to the experimental condition and that to the control condition (Gusnard and Raichle, 2001). Accordingly, a greater relative response to the control condition or a greater relative decrease in activation to the experimental condition could result in a hypoactivation in this region at the individual level. Adding a group variable results in further comparison between individual-level contrasts, complicating the understanding of what the group-level result may reflect (Gusnard and Raichle, 2001). This issue can be addressed by directly assessing activation to the neutral condition (e.g., neutral > fixation cross baseline contrast). In chronic schizophrenia patients, a recent meta-analysis of brain activation to neutral stimuli compared to a baseline condition found increased amygdala responsivity compared to healthy controls (Dugré et al., 2019). In our systematic review, only two of eleven eligible studies assessed a neutral > baseline contrast in FEP patients and found inconsistent results which did not involve the amygdala. As such, this limited number of studies precludes addressing the hypothesis of whether amygdala hypoactivation, measurable at first psychosis episode, is driven by an underlying hyperresponse to the neutral comparator condition. This is of special interest, as evidence from post-mortem and preclinical research suggest increased amygdala reactivity in schizophrenia may be due to either increased activity of a local feedforward excitation circuit (Benes, 2010) or decreased regulation from the prefrontal cortex (Aleman and Kahn, 2005), which could be concomitant to decreased GABA-synthesising enzyme GAD67 function in the hippocampus (Benes, 2010). Indeed, amygdala hyperresponsivity has been shown in a neurodevelopmental model of psychosis, in which a functional loss of parvalbumin interneurons in the hippocampus is associated with increased dopaminergic activity in the striatum (Lodge et al., 2009; Lodge and Grace, 2007; Heckers and Konradi, 2015). On the other hand, behavioural meta-analyses showed a more consistent impairment in emotional but not neutral facial expression recognition in FEP and schizophrenia (Barkl et al., 2014; Bosnjak Kuharic et al., 2019). Together with the current understanding of the role of the amygdala in salience identification, these findings would suggest lower amygdala recruitment during the viewing of emotional stimuli in psychosis. Moreover, decreased amygdala activation to emotional stimuli and increased activation to neutral stimuli are not mutually exclusive (Aleman and Kahn, 2005). If both were present, an emotion > neutral contrast would show relatively lower activation to emotional stimuli, just as it would if only response to emotional stimuli was lower but response to neutral was unchanged, or if response to neutral stimuli was heightened but that to emotional stimuli was unchanged (Gusnard and Raichle, 2001). Furthermore, either of the three response patterns could be present in different individuals and collectively show as amygdala hypoactivation, as it was shown that paranoid but not nonparanoid schizophrenia patients show increased baseline amygdala perfusion (Pinkham et al., 2015). Future studies directly assessing activation to the neutral condition in FEP patients and healthy controls are needed to better characterise the role of the amygdala in psychosis expression.

Hypoactivation of the ACC to emotional stimuli in FEP patients was also a consistent finding in both our systematic review and the meta-analysis, converging with previous meta-analytic findings in schizophrenia (Taylor et al., 2012; Dong et al., 2018). Similarly as for the amygdala, the inconsistency with a previous meta-analysis may derive from the different methodological approaches applied (Del Casale et al., 2018; Radua et al., 2012). The ACC has been implicated in facial emotion stimuli processing (Murphy et al., 2003; Lindquist et al., 2012; Vytal and Hamann, 2010; Kirby and Robinson, 2017), the most predominantly used stimulus type across studies. The ACC receives strong projections from the amygdala (Benes, 2010), supporting the involvement of these two regions in emotion processing. An automated meta-analysis of fMRI studies in healthy controls found that, in contrast to the amygdala, the ACC was activated across paradigms involving emotion, memory and pain stimuli and its recruitment was not selective for emotion in a reverse inference analysis (Yarkoni et al., 2011). Moreover, ACC lesions are associated with various affective disorders including anxiety and apathy (Phillips et al., 2003), and ACC activation has been associated with internal state monitoring (Pace-Schott et al., 2019). Decreased (Zhou et al., 2019) or absent (de la Fuente-Sandoval et al., 2012) ACC response during pain processing in patients with schizophrenia compared to healthy controls has also been reported, supporting the view that its role spans beyond emotion processing. Since ACC subdivisions were associated with various functions (e.g., rostral ACC in emotional paradigms more than its dorsal subdivision), ACC subdivision volume reductions in schizophrenia may reflect functional segregation (Fornito et al., 2009). However, a meta-analysis of structural neuroimaging studies in FEP reported no significant differences in ACC volume relative to healthy controls (Fusar-Poli et al., 2012), suggesting the potential involvement of more subtle, functional changes such as functional connectivity abnormalities of this region (Li et al., 2017; Gong et al., 2017). Taken together, the evidence above suggests that while abnormal amygdala recruitment in psychosis may be specifically linked to emotion processing abnormalities, ACC hypoactivation may be reflective of a more generalised affective abnormality, potentially involving interoceptive processes.

Several other findings from the studies on FEP patients in regions associated with emotion processing are of note. Decreased response to emotion in the lingual gyrus (Tseng et al., 2016; Yang and Wang, 2017; Bergé et al., 2014; Knolle et al., 2018) and the medial frontal gyrus/medial prefrontal cortex (Bergé et al., 2014; Das et al., 2007; Hempel et al., 2003; Reske et al., 2009; Catalucci et al., 2011; Ebisch et al., 2013) in FEP patients was reported by several articles. These results were confirmed by meta-analysis. As both prefrontal and occipital regions have been implicated in emotion processing in healthy volunteers (Vytal and Hamann, 2010; Kirby and Robinson, 2017; Fusar-Poli et al., 2009), our findings suggest altered involvement of such regions in emotion processing in FEP groups. Finally, the insula is also commonly associated with emotion processing (Murphy et al., 2003; Vytal and Hamann, 2010; Kirby and Robinson, 2017; Fusar-Poli et al., 2009). In FEP, decreases in this region/frontal operculum were reported in three articles (Bergé et al., 2014; Knolle et al., 2018; Catalucci et al., 2011), and increases by another two (Reske et al., 2009; Ebisch et al., 2013). The meta-analysis could only include one of those studies and returned significant bilateral hypoactivation in this region. Overall, the results suggest prefrontal, occipital and insula involvement in the pathophysiology of emotion alterations in FEP patients.

In people at CHRp, the only convergent finding was of increased PCC response to emotion, reported by two reviewed studies including facial stimuli (Derntl et al., 2015; Van Der Velde et al., 2015). This was also identified by a recent review on social cognition deficits in CHRp (Kozhuharova et al., 2019), suggesting this area may be overly responsive to socially-relevant emotional cues. Interestingly, two studies found increased activation in the inferior frontal gyrus to the neutral condition in the CHRp group (Modinos et al., 2015; Seiferth et al., 2008). Hyperresponsivity to the neutral condition was also reported by another publication, but in the left temporal pole and bilateral PCC (Van Der Velde et al., 2015). Since all three studies used different methods for analysing the neutral condition (comparison of activation parameters to the neutral condition (Seiferth et al., 2008), neutral > fixation cross comparison (Modinos et al., 2015) and neutral > blank screen comparison (Van Der Velde et al., 2015)), more evidence is needed to characterise neural response to neutral stimuli in CHRp. Furthermore, none of these findings were supported by meta-analysis, and we identified no other convergent results between CHRp studies.

The absence of effects surviving p_TFCE_ < 0.05 correction from our meta-analysis on CHRp studies could be due to more task paradigm-specific responses in this group, or heterogeneity of the samples included. About 26 % of people at CHRp transition to frank psychosis within two years of initial assessment (Fusar-Poli et al., 2015b), therefore samples comprise individuals with a variety of outcomes. In addition, CHRp status is not a formal diagnosis but a collection of three potentially concurrent at-risk criteria spanning a wide symptomatic continuum and/or genetic factors (Yung et al., 2005). Of the studies reviewed, only one (Gee et al., 2012) reported the CHRp sub-type composition of their sample. Future studies reporting this will expand our understanding of emotional responsivity according to CHRp subtype and clarify whether this is a potential source of heterogeneity in imaging findings. Finally, people at CHRp often have a formal diagnosis of another Axis I disorder, such as anxiety or depression (Shi et al., 2017; Fusar-Poli et al., 2014), and the potential concomitant use of antidepressant or anxiolytic medication may have an effect on the imaging data in response to emotion stimuli (Murphy et al., 2009; Paulus et al., 2005). More consistent task paradigm, stimuli and CHRp assessment criteria across studies would aid understanding of the nature of emotion-related neuroimaging abnormalities in the CHRp state.

Overall, the effects of antipsychotic medication on the results remain unclear. Antipsychotics have been shown to have no effects on emotion perception in chronic patients (Penn et al., 2009). In the present review, one paper (Bergé et al., 2014) re-examined the antipsychotic-naïve FEP group upon clinical improvement with antipsychotic treatment after 3–6 weeks and found no differences in brain activation to emotion of FEP patients to HCs. However, a separate study found predominantly stable fMRI activation abnormalities during sad and happy mood induction in medicated FEP patients (Reske et al., 2007). Moreover, four of the studies reviewed (Tseng et al., 2016; Das et al., 2007; Ebisch et al., 2013; Modinos et al., 2017) regressed medication status onto fMRI signal and found no effect. Hence, although current evidence of antipsychotic medication effects on the neural basis of emotion processing in FEP remains to be determined, the evidence suggests that it is likely to have negligible effects on the fMRI results reviewed. This may indicate a need for more targeted treatments for emotion processing deficits in these groups to improve functional and clinical outcomes.

There are some limitations to the current work. Firstly, the number of t-maps made available for the meta-analyses was small. However, the SDM validation process indicated that three raw images were the minimum to reach 100 % sensitivity on the analysis, exceeding that of coordinate-based methods (Albajes-Eizagirre et al., 2019). Furthermore, we assessed the heterogeneity of the results obtained for the FEP meta-analysis and found no variation attributable to study heterogeneity in the peaks reported in Results. Hence, despite generalisability may be limited, the present results can be considered reliable. Moreover, as the first such meta-analysis of its kind, future greater availability of data is warranted. The meta-analytic results were also supported by a systematic review, where results from all studies identified were synthesised, thus avoiding selection bias e.g., due to inclusion of results within pre-specified brain regions only. Secondly, some task and sample variability in the studies included was present. More specifically, auditory stimuli were used by two studies (Quarmley et al., 2019; Tseng et al., 2016), and the age range of CHRp participants was wider than that of FEP participants, with two studies including adolescents (Quarmley et al., 2019; Gee et al., 2012). However, this approach may be more ecologically valid, and overall through our stringent clinical inclusion criteria we ensured that the analysed samples were as homogenous as possible to increase the clinical validity of the review. Due to two out of three datasets analysed including antipsychotic-naïve patients with an FEP, we could not perform a meta-regression assessing antipsychotic medication status on the results of the meta-analysis. However, the systematic review indicated that most studies found no effects of antipsychotic medication on the results, and the samples analysed were mostly antipsychotic-naïve or free. Regrettably, we could not assess the putative influence of task design on meta-analysis results (Li et al., 2010) due to the limited number of studies employing an implicit task paradigm (Table 1). Finally, because two of the studies that included both FEP and CHRp participants had used the same control group, we could not reliably perform a combined meta-analysis of FEP + CHRp t-maps. In the context of a combined meta-analysis, this shared control group would be treated as two independent groups, which could lead to spurious results as in fact these were the same individuals. Nevertheless, for illustrative purposes we include such analysis in the Supplement (Supplementary Methods, Supplementary Results and Figure S2).

In conclusion, while widespread hypoactivations to emotional compared to neutral stimuli were reported and corroborated by meta-analysis for FEP studies, the inconsistency of neuroimaging results in CHRp studies was matched by no significant effects in the corresponding meta-analysis. Three questions arise from this work to be addressed in future studies. Firstly, the nature of hypoactivations in FEP remains to be clarified, especially in the context of the neutral comparison condition. Secondly, more consistency in CHRp participant inclusion and imaging paradigms would help elucidate whether abnormal fMRI response to emotion is present and detectable in this group. Finally, greater raw data availability will help expand the present findings and this can be facilitated through data upload on open source depositories. These efforts will help elucidate the role of emotion abnormalities in the pathophysiology of psychosis risk and onset and inform the development of much-needed treatments for psychosis symptomatology beyond positive symptoms.

## Funding

This research did not receive any specific grant from funding agencies in the public, commercial, or not-for-profit sectors. Paulina Lukow is in receipt of a PhD studentship funded by the National Institute for Health Research (NIHR) Biomedical Research Centre at South London and Maudsley NHS Foundation Trust and King’s College London. The views expressed are those of the author(s) and not necessarily those of the NHS, the NIHR or the Department of Health and Social Care. Gemma Modinos is funded by a Sir Henry Dale Fellowship jointly funded by the 10.13039/100004440Wellcome Trust and the 10.13039/501100000288Royal Society (#202397/Z/16/Z).

## Declaration of Competing Interest

The authors report no declarations of interest.
