# Supplementary material for: Neural correlates of emotional processing in psychosis risk and onset – A systematic review and meta-analysis of fMRI studies
Source: Neurosci Biobehav Rev. 2021 Sep;128:780–8. doi: 10.1016/j.neubiorev.2021.03.010 (PMC8345001; doi:10.1016/j.neubiorev.2021.03.010)
Supplement: Supplementary file 1 [file mmc1.docx]

SUPPLEMENTARY MATERIAL

Neural correlates of emotional processing in psychosis risk and onset – a systematic review and meta-analysis of fMRI studies

LUKOW PB*^a^, Kiemes A^a^, Kempton MJ^a^, Turkheimer FE^b^, McGuire P^a^, Modinos G^a,b,c^

^a^Department of Psychosis Studies, Institute of Psychiatry, Psychology & Neuroscience, King’s College London, De Crespigny Park, SE5 8AF, London, UK

^b^Department of Neuroimaging, Institute of Psychiatry, Psychology & Neuroscience, King’s College London, De Crespigny Park, SE5 8AF, London, UK

^c^MRC Centre for Neurodevelopmental Disorders, King's College London, New Hunt's House, Guy's Campus, SE1 1UL, London, UK

*Corresponding author: paulina.lukow@kcl.ac.uk

Other authors’ contact email addresses: amanda.s.kiemes@kcl.ac.uk, matthew.kempton@kcl.ac.uk, federico.turkheimer@kcl.ac.uk, philip.mcguire@kcl.ac.uk, gemma.modinos@kcl.ac.uk

**
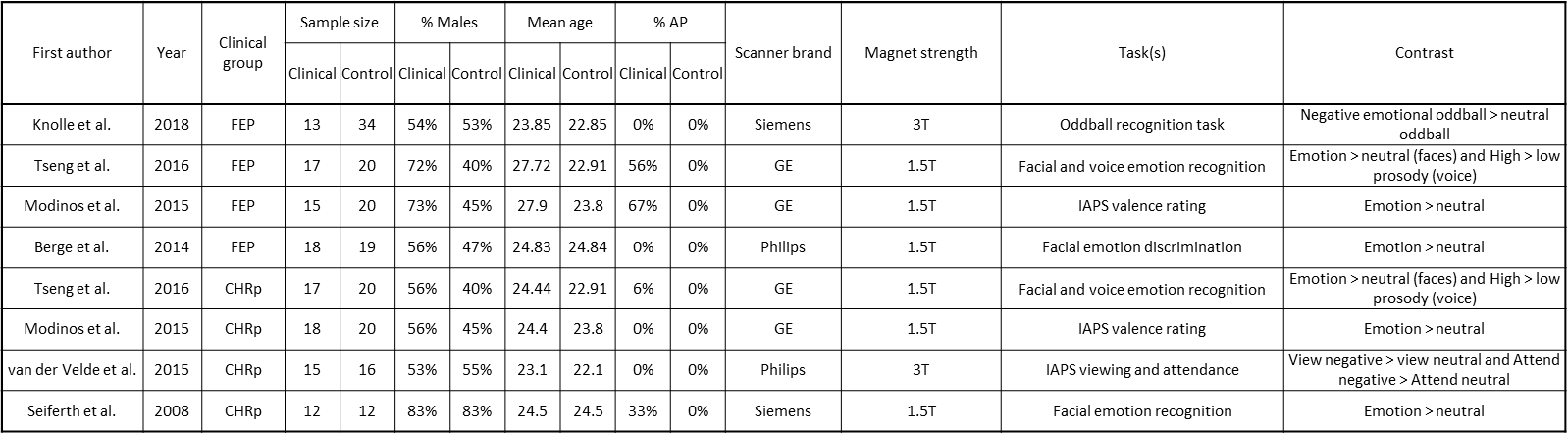
Table S1. Methodological aspects of studies meta-analysed.**

%AP, percentage of participants on antipsychotic medication. FEP, first episode of psychosis. CHRp, clinical high-risk for psychosis.

Demographic details of the FEP and CHRp samples are reported separately for studies including both groups.

**Uniformity of studies included – FEP**

Nine studies used facial emotion expressions as stimuli (1–9). One study additionally used pictures and words from the International Affective Picture System (IAPS) set (1), one used outdoor scenes (9) and another one applied a second task with emotional prosody recognition (2). Three studies contained an emotion recognition (labelling) task, and all others used a different and unique paradigm such as emotion matching or discrimination (Table 1). Of the remaining two studies, one employed observation of scenes of touch by an inanimate object or another person, the latter with different valence, without the viewing of involved persons’ face (10). The remaining study utilised highly disgusting images, as well as highly pleasant stimuli such as small animals, food and wounds (11).

Overall, 10 studies included a non-emotional, neutral condition in their experiment (1–10) and 6 used it as comparison for the emotional condition (1–3,5,7,9). More specifically, between-group contrasts involved (1) several combined emotional valences > neutral (1,2,8), (2) a conjunction analysis of several emotional valences > neutral (10), (3) a single emotional valence > neutral (5,8,9,11), (4) a group*task ANOVA (3,4), (5) a single emotional valence recognition (implicit/explicit) > gender discrimination (7), or (6) all emotional valences across the task performed (6).

Four studies included antipsychotic-naïve patients only (4,8,9,11). One of these re-scanned patients at post-treatment follow-up (8). Three studies included only antipsychotic-medicated patients into their study (3,6,7). The remaining 4 studies included both medication-naïve patients and those taking antipsychotic medication (1,2,5,10).

**Uniformity of studies included – CHRp**

The most common paradigm was facial emotion recognition (2,12–14), whereby the participant is asked to label the emotional expression being presented. Three of them also used a neutral condition for comparison (2,12,14). Tseng et al. additionally used a task with prosodic emotional stimuli of high or low intensity (2). One other study used neutral facial stimuli, however the paradigm involved fear conditioning with a scream (15). Two studies used the International Affective Picture System (IAPS, (16)); one asked participants to rate their subjective emotional arousal to negative or positive stimuli of high or low arousal (1), while the other included conditions of viewing neutral images and/or reappraisal of negative ones (17).

Between-group contrasts involved (1) several combined emotional valences > neutral (1,2), (2) a conjunction analysis of several emotional > neutral (12), (3) a single emotional valence > neutral (15,17), (4) a group*task ANOVA (14), or (5) emotional valence labelling > gender labelling/emotion matching (13).

Participants included in the above studies were predominantly free of any psychoactive medication, although only one study included antipsychotic-free individuals (1). Two studies included participants on antipsychotic medication (2,15), three studies additionally allowed antidepressant medication (12–14) and the remaining study included participants also on psychostimulant medication (17).

**Search terms for the Ovid MEDLINE database. The same search terms were used for the Pubmed interface but restricted to title and abstract. No other restrictions were placed.**

("clinical high" OR "ultra high" OR "at risk" OR "risk" OR transition* OR "high risk" OR prodrom* OR "attenuated" OR "ultra-high-risk" OR "clinical-high-risk" OR "CHR" OR "UHR" OR "first" OR "episode" OR "spectrum" OR "early" OR "onset" OR "first-onset") AND ("psychosis" OR psychot* OR schizophren* OR "mental state") AND ("limbic" OR emotion* OR "mPFC" OR "medial prefrontal cortex" OR "medial PFC" OR amygdal* OR "BLA" OR hippocamp* OR "ACC" OR "ACG" OR "anterior cingulate" OR "orbitofrontal" OR "insula") AND ("limbic" OR emotion* OR "mPFC" OR "medial prefrontal cortex" OR "medial PFC" OR amygdal* OR "BLA" OR hippocamp* OR "ACC" OR "ACG" OR "anterior cingulate" OR "orbitofrontal" OR "insula") AND ("PET" OR "positron emission tomography" OR "SPECT" OR "single photon emission computed tomography" OR "MRI" OR "fMRI" OR "functional magnetic resonance imaging" OR "PET/MR" OR "PET/MRI" OR "voxel" OR "BOLD" OR "blood-oxygen-level-dependent imaging" OR "perfusion" OR "arterial spin" OR "ASL" OR "magnetic resonance spectroscopy" OR "MRS" OR function*)

**The PRISMA checklist for reporting the systematic review and meta-analysis**

| **Section/topic** | **#** | **Checklist item** | **Reported on page #** |
| --- | --- | --- | --- |
| **TITLE** | | |  |
| Title | 1 | Identify the report as a systematic review, meta-analysis, or both. | 1 |
| **ABSTRACT** | | |  |
| Structured summary | 2 | Provide a structured summary including, as applicable: background; objectives; data sources; study eligibility criteria, participants, and interventions; study appraisal and synthesis methods; results; limitations; conclusions and implications of key findings; systematic review registration number. | 2 |
| **INTRODUCTION** | | |  |
| Rationale | 3 | Describe the rationale for the review in the context of what is already known. | 3-5 |
| Objectives | 4 | Provide an explicit statement of questions being addressed with reference to participants, interventions, comparisons, outcomes, and study design (PICOS). | 5-6 |
| **METHODS** | | |  |
| Protocol and registration | 5 | Indicate if a review protocol exists, if and where it can be accessed (e.g., Web address), and, if available, provide registration information including registration number. | NA* |
| Eligibility criteria | 6 | Specify study characteristics (e.g., PICOS, length of follow-up) and report characteristics (e.g., years considered, language, publication status) used as criteria for eligibility, giving rationale. | 5-6 |
| Information sources | 7 | Describe all information sources (e.g., databases with dates of coverage, contact with study authors to identify additional studies) in the search and date last searched. | 5 |
| Search | 8 | Present full electronic search strategy for at least one database, including any limits used, such that it could be repeated. | Supplement |
| Study selection | 9 | State the process for selecting studies (i.e., screening, eligibility, included in systematic review, and, if applicable, included in the meta-analysis). | 6-7 |
| Data collection process | 10 | Describe method of data extraction from reports (e.g., piloted forms, independently, in duplicate) and any processes for obtaining and confirming data from investigators. | 6 |
| Data items | 11 | List and define all variables for which data were sought (e.g., PICOS, funding sources) and any assumptions and simplifications made. | 6 |
| Risk of bias in individual studies | 12 | Describe methods used for assessing risk of bias of individual studies (including specification of whether this was done at the study or outcome level), and how this information is to be used in any data synthesis. | 6 |
| Summary measures | 13 | State the principal summary measures (e.g., risk ratio, difference in means). | NA** |
| Synthesis of results | 14 | Describe the methods of handling data and combining results of studies, if done, including measures of consistency (e.g., I^2^) for each meta-analysis. | 7-8 |
| Risk of bias across studies | 15 | Specify any assessment of risk of bias that may affect the cumulative evidence (e.g., publication bias, selective reporting within studies). | NA*** |
| Additional analyses | 16 | Describe methods of additional analyses (e.g., sensitivity or subgroup analyses, meta-regression), if done, indicating which were pre-specified. | NA**** |
| **RESULTS** | | |  |
| Study selection | 17 | Give numbers of studies screened, assessed for eligibility, and included in the review, with reasons for exclusions at each stage, ideally with a flow diagram. | 8-9 and Figure 1 |
| Study characteristics | 18 | For each study, present characteristics for which data were extracted (e.g., study size, PICOS, follow-up period) and provide the citations. | 6-8 |
| Risk of bias within studies | 19 | Present data on risk of bias of each study and, if available, any outcome level assessment (see item 12). | 8 |
| Results of individual studies | 20 | For all outcomes considered (benefits or harms), present, for each study: (a) simple summary data for each intervention group (b) effect estimates and confidence intervals, ideally with a forest plot. | NA** |
| Synthesis of results | 21 | Present results of each meta-analysis done, including confidence intervals and measures of consistency. | 10-11 and 12 |
| Risk of bias across studies | 22 | Present results of any assessment of risk of bias across studies (see Item 15). | NA*** |
| Additional analysis | 23 | Give results of additional analyses, if done (e.g., sensitivity or subgroup analyses, meta-regression [see Item 16]). | NA**** |
| **DISCUSSION** | | |  |
| Summary of evidence | 24 | Summarize the main findings including the strength of evidence for each main outcome; consider their relevance to key groups (e.g., healthcare providers, users, and policy makers). | 13-17 |
| Limitations | 25 | Discuss limitations at study and outcome level (e.g., risk of bias), and at review-level (e.g., incomplete retrieval of identified research, reporting bias). | 17-18 |
| Conclusions | 26 | Provide a general interpretation of the results in the context of other evidence, and implications for future research. | 18-19 |
| **FUNDING** | | |  |
| Funding | 27 | Describe sources of funding for the systematic review and other support (e.g., supply of data); role of funders for the systematic review. | 19 |

*From:*  Moher D, Liberati A, Tetzlaff J, Altman DG, The PRISMA Group (2009). Preferred Reporting Items for Systematic Reviews and Meta-Analyses: The PRISMA Statement. PLoS Med 6(7): e1000097. doi:10.1371/journal.pmed1000097

For more information, visit: **www.prisma-statement.org**.

* The protocol for this systematic review was not pre-registered.

** Original group comparison t-maps were used instead of reported results.

*** Due to the low numbers of studies included in each meta-analysis, risk of bias across studies was not performed.

**** No additional analyses were performed.

**Table S2. Local peak details of the meta-analysis of brain activation to emotion in FEP patients versus healthy controls. Results are shown for the peak with the largest Z value for defined brain regions, pTFCE < 0.05.**

| Local peaks: |  |  |  |
| --- | --- | --- | --- |
| *MNI coordinate* | *SDM-Z* | *P* | Description |
| -6,-76,6 | -5.284 | 0.000999987 | Left inferior network, inferior longitudinal fasciculus |
| 6,-72,2 | -5.243 | 0.000999987 | Right lingual gyrus, BA 17 |
| -4,-84,8 | -5.073 | 0.000999987 | Left calcarine fissure / surrounding cortex, BA 18 |
| 2,-72,20 | -4.924 | 0.000999987 | Left cuneus cortex, BA 18 |
| 10,-76,2 | -4.857 | 0.000999987 | Right inferior network, inferior longitudinal fasciculus |
| 10,-70,16 | -4.711 | 0.000999987 | (undefined) |
| 28,2,-12 | -4.669 | 0.000999987 | (undefined), BA 34 |
| -38,2,-10 | -4.655 | 0.000999987 | Left insula, BA 48 |
| -8,-52,-6 | -4.62 | 0.000999987 | Left cerebellum, hemispheric lobule IV / V, BA 18 |
| 22,-58,2 | -4.604 | 0.000999987 | Corpus callosum |
| 26,-62,6 | -4.521 | 0.000999987 | Right calcarine fissure / surrounding cortex, BA 19 |
| 66,-24,-6 | -4.434 | 0.000999987 | Right middle temporal gyrus, BA 21 |
| 4,-82,16 | -4.364 | 0.000999987 | Right cuneus cortex, BA 18 |
| 6,-44,-6 | -4.364 | 0.000999987 | Cerebellum, vermic lobule IV / V |
| 54,6,-2 | -4.286 | 0.000999987 | Right temporal pole, superior temporal gyrus, BA 38 |
| 2,2,40 | -4.205 | 0.000999987 | Right median cingulate / paracingulate gyri, BA 24 |
| -12,-66,-6 | -4.201 | 0.000999987 | Left lingual gyrus, BA 18 |
| 62,-38,2 | -4.18 | 0.000999987 | Right middle temporal gyrus, BA 22 |
| 54,0,-12 | -4.151 | 0.000999987 | Right superior temporal gyrus, BA 21 |
| -18,-28,-6 | -4.13 | 0.000999987 | Left optic radiations |
| -48,-30,8 | -4.129 | 0.000999987 | Left superior temporal gyrus, BA 48 |
| 16,-44,-12 | -4.112 | 0.000999987 | Right cerebellum, hemispheric lobule IV / V, BA 30 |
| -18,-10,-12 | -4.093 | 0.000999987 | Left hippocampus |
| -30,6,-16 | -4.056 | 0.000999987 | (undefined), BA 38 |
| 2,52,-10 | -4.028 | 0.000999987 | Right superior frontal gyrus, medial orbital, BA 11 |
| -26,-2,-14 | -4.027 | 0.000999987 | Left amygdala, BA 34 |
| 14,-12,-12 | -4.017 | 0.000999987 | Right cortico-spinal projections |
| 54,-12,8 | -4.007 | 0.000999987 | Right heschl gyrus, BA 48 |
| 56,-14,-8 | -3.99 | 0.000999987 | Right superior temporal gyrus, BA 22 |
| 10,-10,-12 | -3.985 | 0.000999987 | Right pons |
| 28,-38,4 | -3.95 | 0.000999987 | Right hippocampus, BA 37 |
| 12,-60,30 | -3.933 | 0.000999987 | Right median network, cingulum |
| 24,-34,4 | -3.93 | 0.000999987 | Right hippocampus, BA 27 |
| 30,24,-6 | -3.907 | 0.000999987 | Right inferior frontal gyrus, orbital part, BA 47 |
| -14,-48,-4 | -3.901 | 0.000999987 | Left lingual gyrus, BA 30 |
| 54,-52,-6 | -3.887 | 0.000999987 | Right inferior temporal gyrus, BA 21 |
| 2,-72,28 | -3.869 | 0.000999987 | Left cuneus cortex |
| 38,-18,12 | -3.861 | 0.000999987 | Right insula, BA 48 |
| 24,-16,-14 | -3.83 | 0.000999987 | Right hippocampus |
| -12,-16,36 | -3.822 | 0.000999987 | Left median network, cingulum |
| 52,-4,0 | -3.812 | 0.000999987 | Right superior temporal gyrus, BA 48 |
| -22,8,-8 | -3.802 | 0.000999987 | Left lenticular nucleus, putamen, BA 48 |
| -34,18,-12 | -3.801 | 0.000999987 | Left insula, BA 47 |
| -14,-64,14 | -3.801 | 0.000999987 | Left calcarine fissure / surrounding cortex, BA 17 |
| -50,-50,10 | -3.788 | 0.000999987 | Left middle temporal gyrus, BA 21 |
| 12,-34,0 | -3.774 | 0.000999987 | (undefined), BA 27 |
| -24,-36,-2 | -3.76 | 0.000999987 | Left hippocampus, BA 37 |
| -22,12,-12 | -3.756 | 0.000999987 | Left inferior network, uncinate fasciculus |
| 34,18,-14 | -3.74 | 0.000999987 | Right insula, BA 47 |
| 8,-18,2 | -3.737 | 0.000999987 | Right anterior thalamic projections |
| -10,-12,42 | -3.723 | 0.000999987 | Left median cingulate / paracingulate gyri |
| 12,-30,-6 | -3.707 | 0.000999987 | Right lingual gyrus, BA 27 |
| 6,-66,30 | -3.695 | 0.000999987 | Right precuneus |
| 0,-10,42 | -3.674 | 0.000999987 | Left median cingulate / paracingulate gyri, BA 23 |
| -42,-20,6 | -3.635 | 0.000999987 | Left heschl gyrus, BA 48 |
| 48,20,-6 | -3.633 | 0.000999987 | Right inferior frontal gyrus, orbital part |
| -18,-36,0 | -3.624 | 0.000999987 | Left hippocampus, BA 27 |
| 6,-50,22 | -3.614 | 0.000999987 | Right precuneus, BA 23 |
| 8,-48,34 | -3.614 | 0.000999987 | Right median cingulate / paracingulate gyri, BA 23 |
| 0,40,4 | -3.611 | 0.000999987 | Left anterior cingulate / paracingulate gyri |
| -50,0,2 | -3.608 | 0.000999987 | Left rolandic operculum, BA 48 |
| 44,8,40 | -3.604 | 0.000999987 | Right middle frontal gyrus, BA 6 |
| 46,-50,22 | -3.594 | 0.000999987 | Right angular gyrus, BA 41 |
| -42,-72,14 | -3.565 | 0.000999987 | Left middle occipital gyrus, BA 37 |
| 46,4,-10 | -3.533 | 0.000999987 | Right insula |
| -12,-18,0 | -3.495 | 0.000999987 | Left anterior thalamic projections |
| 6,-26,6 | -3.463 | 0.000999987 | Right thalamus |
| 44,-40,4 | -3.446 | 0.000999987 | Right arcuate network, posterior segment |
| 32,-18,-16 | -3.444 | 0.000999987 | Right hippocampus, BA 20 |
| 24,12,-6 | -3.438 | 0.000999987 | Right lenticular nucleus, putamen, BA 48 |
| 20,-56,14 | -3.432 | 0.000999987 | Right precuneus, BA 17 |
| 54,-60,-8 | -3.404 | 0.000999987 | Right inferior temporal gyrus, BA 37 |
| 30,-14,-4 | -3.366 | 0.000999987 | Right striatum |
| -8,54,-8 | -3.356 | 0.000999987 | Left superior frontal gyrus, medial orbital, BA 11 |
| 46,-8,46 | -3.354 | 0.000999987 | Right precentral gyrus, BA 6 |
| 34,10,4 | -3.35 | 0.000999987 | (undefined), BA 48 |
| 40,16,-14 | -3.348 | 0.000999987 | Right insula, BA 38 |
| -58,-42,14 | -3.347 | 0.000999987 | Left superior temporal gyrus, BA 42 |
| 34,-88,-2 | -3.338 | 0.000999987 | Right inferior occipital gyrus, BA 19 |
| 34,10,26 | -3.322 | 0.000999987 | Right superior longitudinal fasciculus III |
| -58,-24,6 | -3.286 | 0.000999987 | Left superior temporal gyrus, BA 22 |
| -36,26,-6 | -3.279 | 0.000999987 | Left inferior frontal gyrus, orbital part, BA 47 |
| -22,-62,16 | -3.264 | 0.000999987 | (undefined), BA 17 |
| -52,4,-6 | -3.261 | 0.000999987 | Left superior temporal gyrus, BA 38 |
| 62,-10,20 | -3.239 | 0.000999987 | Right postcentral gyrus, BA 43 |
| 42,38,24 | -3.225 | 0.000999987 | Right middle frontal gyrus, BA 45 |
| 56,-2,10 | -3.224 | 0.000999987 | Right rolandic operculum, BA 48 |
| 46,-40,10 | -3.22 | 0.000999987 | Right superior temporal gyrus, BA 41 |
| -8,-68,22 | -3.217 | 0.000999987 | Left calcarine fissure / surrounding cortex, BA 23 |
| 10,-50,8 | -3.207 | 0.000999987 | Right precuneus, BA 29 |
| -12,-50,6 | -3.203 | 0.000999987 | Left calcarine fissure / surrounding cortex, BA 30 |
| 10,46,2 | -3.184 | 0.000999987 | Right anterior cingulate / paracingulate gyri, BA 10 |
| -30,-90,0 | -3.124 | 0.000999987 | Left middle occipital gyrus, BA 18 |
| 14,-46,36 | -3.108 | 0.000999987 | Right median cingulate / paracingulate gyri |
| 16,-10,24 | -3.098 | 0.000999987 | Right caudate nucleus |
| -44,-64,12 | -3.094 | 0.000999987 | Left middle temporal gyrus, BA 37 |
| -2,-18,2 | -3.086 | 0.000999987 | Left thalamus |
| 36,-20,44 | -3.076 | 0.000999987 | Right superior longitudinal fasciculus II |
| -42,-52,16 | -3.063 | 0.000999987 | Left arcuate network, posterior segment |
| 8,-90,2 | -3.059 | 0.000999987 | Right calcarine fissure / surrounding cortex, BA 17 |
| 38,12,24 | -3.059 | 0.000999987 | Right frontal inferior longitudinal fasciculus |
| 10,52,-4 | -3.055 | 0.000999987 | Right superior frontal gyrus, medial orbital, BA 10 |
| 38,8,50 | -3.049 | 0.000999987 | Right middle frontal gyrus, BA 9 |
| -38,32,-4 | -3.018 | 0.000999987 | Left inferior network, inferior fronto-occipital fasciculus |
| -16,22,-2 | -2.983 | 0.000999987 | Left striatum |
| -30,-72,22 | -2.967 | 0.000999987 | Left middle occipital gyrus, BA 19 |
| 24,18,48 | -2.961 | 0.000999987 | Right superior frontal gyrus, dorsolateral, BA 8 |
| -44,16,-6 | -2.949 | 0.000999987 | Left inferior frontal gyrus, orbital part |
| -46,28,0 | -2.945 | 0.000999987 | Left inferior frontal gyrus, triangular part, BA 47 |
| 50,12,32 | -2.921 | 0.000999987 | Right inferior frontal gyrus, opercular part, BA 44 |
| 50,-70,0 | -2.918 | 0.000999987 | Right middle temporal gyrus, BA 37 |
| 8,40,34 | -2.907 | 0.000999987 | Right superior frontal gyrus, medial, BA 32 |
| 30,-10,8 | -2.899 | 0.000999987 | Right fronto-insular tract 5 |
| 4,56,28 | -2.887 | 0.000999987 | Right superior frontal gyrus, medial, BA 10 |
| 50,-56,32 | -2.886 | 0.000999987 | Right angular gyrus, BA 39 |
| 4,48,28 | -2.863 | 0.000999987 | Right anterior cingulate / paracingulate gyri |
| -44,-38,20 | -2.859 | 0.000999987 | Left superior temporal gyrus, BA 41 |
| 0,8,-4 | -2.856 | 0.000999987 | (undefined), BA 25 |
| 24,-2,48 | -2.836 | 0.000999987 | Right frontal superior longitudinal |
| 28,-36,-12 | -2.811 | 0.000999987 | Right parahippocampal gyrus, BA 37 |
| 54,30,2 | -2.793 | 0.000999987 | Right inferior frontal gyrus, triangular part, BA 45 |
| 0,54,12 | -2.785 | 0.000999987 | Left superior frontal gyrus, medial |
| -24,56,-2 | -2.783 | 0.013000011 | Left superior frontal gyrus, orbital part, BA 11 |
| -36,10,38 | -2.772 | 0.004000008 | Left superior longitudinal fasciculus II |
| -46,-8,44 | -2.77 | 0.003000021 | Left precentral gyrus, BA 6 |
| -44,-40,24 | -2.748 | 0.000999987 | Left supramarginal gyrus, BA 41 |
| 0,18,-6 | -2.731 | 0.000999987 | Left olfactory cortex |
| -2,12,-8 | -2.715 | 0.000999987 | Left olfactory cortex, BA 25 |
| -36,12,34 | -2.71 | 0.004000008 | Left middle frontal gyrus, BA 44 |
| 24,10,48 | -2.707 | 0.000999987 | Right middle frontal gyrus, BA 8 |
| 2,30,26 | -2.685 | 0.000999987 | Right anterior cingulate / paracingulate gyri, BA 24 |
| 58,-2,18 | -2.682 | 0.000999987 | Right postcentral gyrus, BA 48 |
| 46,-18,44 | -2.674 | 0.000999987 | Right precentral gyrus, BA 4 |
| -52,12,2 | -2.656 | 0.000999987 | Left inferior frontal gyrus, opercular part, BA 48 |
| 38,24,24 | -2.647 | 0.000999987 | Right inferior frontal gyrus, triangular part, BA 48 |
| -2,60,16 | -2.644 | 0.000999987 | Left superior frontal gyrus, medial, BA 10 |
| -8,6,36 | -2.62 | 0.000999987 | Left median cingulate / paracingulate gyri, BA 24 |
| -16,-6,24 | -2.581 | 0.000999987 | Left caudate nucleus |
| -50,-12,32 | -2.551 | 0.003000021 | Left postcentral gyrus, BA 3 |
| -42,-42,16 | -2.529 | 0.000999987 | Left superior longitudinal fasciculus III |
| 52,-26,42 | -2.489 | 0.000999987 | Right postcentral gyrus, BA 3 |
| 38,-42,38 | -2.462 | 0.029999971 | (undefined), BA 40 |
| -50,-6,28 | -2.46 | 0.003000021 | Left precentral gyrus, BA 4 |
| 44,6,30 | -2.439 | 0.000999987 | Right precentral gyrus, BA 44 |
| 52,-40,38 | -2.433 | 0.032999992 | Right supramarginal gyrus, BA 40 |
| 0,38,20 | -2.43 | 0.000999987 | Left anterior cingulate / paracingulate gyri, BA 24 |
| -40,-78,-4 | -2.422 | 0.000999987 | Left inferior occipital gyrus, BA 19 |
| 26,38,30 | -2.416 | 0.000999987 | Right middle frontal gyrus, BA 46 |
| -56,12,24 | -2.405 | 0.004999995 | Left inferior frontal gyrus, opercular part, BA 44 |
| -28,42,24 | -2.405 | 0.009000003 | Left middle frontal gyrus, BA 46 |
| -8,14,6 | -2.402 | 0.000999987 | Left caudate nucleus, BA 25 |
| -2,36,38 | -2.362 | 0.000999987 | Left superior frontal gyrus, medial, BA 9 |
| 30,60,6 | -2.351 | 0.003000021 | Right middle frontal gyrus, BA 10 |
| 14,48,36 | -2.33 | 0.000999987 | Right superior frontal gyrus, dorsolateral, BA 9 |
| -2,24,46 | -2.311 | 0.000999987 | Left supplementary motor area, BA 8 |
| -10,44,14 | -2.306 | 0.000999987 | Left anterior cingulate / paracingulate gyri, BA 32 |
| 22,48,32 | -2.3 | 0.000999987 | Right middle frontal gyrus |
| -48,-18,42 | -2.288 | 0.003000021 | Left postcentral gyrus, BA 4 |
| -2,32,42 | -2.287 | 0.000999987 | Left superior frontal gyrus, medial, BA 8 |
| -18,-36,38 | -2.283 | 0.000999987 | Left superior longitudinal fasciculus I |
| 4,22,-4 | -2.238 | 0.000999987 | Right olfactory cortex, BA 25 |
| 50,-68,20 | -2.237 | 0.000999987 | Right middle temporal gyrus, BA 39 |
| -50,-60,18 | -2.217 | 0.000999987 | Left middle temporal gyrus, BA 39 |
| 26,58,0 | -2.201 | 0.003000021 | Right superior frontal gyrus, dorsolateral, BA 11 |
| -40,12,24 | -2.16 | 0.004999995 | Left frontal inferior longitudinal fasciculus |
| -42,-16,42 | -2.157 | 0.003000021 | Left postcentral gyrus, BA 6 |
| -26,22,44 | -2.045 | 0.032999992 | Left middle frontal gyrus, BA 9 |
| 24,58,14 | -2.004 | 0.003000021 | Right superior frontal gyrus, dorsolateral, BA 10 |
| 58,20,14 | -1.986 | 0.000999987 | Right inferior frontal gyrus, opercular part, BA 48 |
| 22,56,20 | -1.976 | 0.001999974 | Right superior frontal gyrus, dorsolateral, BA 46 |
| -22,-78,-26 | -1.95 | 0.000999987 | Left cerebellum, crus I |
| 8,-70,-20 | -1.95 | 0.000999987 | Right cerebellum, hemispheric lobule VI |
| 8,-82,-30 | -1.95 | 0.000999987 | Right cerebellum, crus II |
| -24,-62,-34 | -1.95 | 0.000999987 | Left cerebellum, hemispheric lobule VI |
| -2,-74,-22 | -1.95 | 0.000999987 | Cerebellum, vermic lobule VII |
| -4,-84,-16 | -1.95 | 0.000999987 | Left cerebellum, crus I, BA 17 |
| 32,-66,-20 | -1.95 | 0.000999987 | Right cerebellum, hemispheric lobule VI, BA 19 |
| -24,-44,-18 | -1.95 | 0.001999974 | Left fusiform gyrus, BA 37 |
| 16,-70,-22 | -1.95 | 0.000999987 | Right cerebellum, hemispheric lobule VI, BA 18 |
| 34,-56,-28 | -1.95 | 0.000999987 | Right cerebellum, hemispheric lobule VI, BA 37 |
| -30,-66,-22 | -1.95 | 0.000999987 | Left cerebellum, hemispheric lobule VI, BA 19 |
| 0,-70,-16 | -1.95 | 0.000999987 | Cerebellum, vermic lobule VI |
| -16,-70,-22 | -1.95 | 0.000999987 | Left cerebellum, hemispheric lobule VI, BA 18 |
| -12,-76,-34 | -1.95 | 0.000999987 | Left cerebellum, crus II |
| -32,-16,-18 | -1.95 | 0.000999987 | Left hippocampus, BA 20 |
| -30,-70,-14 | -1.95 | 0.000999987 | Left fusiform gyrus, BA 19 |
| 30,-60,-34 | -1.95 | 0.000999987 | Right cerebellum, crus I |
| 28,-76,-28 | -1.949 | 0.000999987 | Right cerebellum, crus I, BA 19 |
| 10,-82,-22 | -1.949 | 0.000999987 | Right cerebellum, crus I, BA 18 |
| -10,-84,-18 | -1.949 | 0.000999987 | Left cerebellum, crus I, BA 18 |
| -26,18,48 | -1.948 | 0.032999992 | Left middle frontal gyrus, BA 8 |
| -16,-54,-12 | -1.948 | 0.000999987 | Left cerebellum, hemispheric lobule IV / V, BA 19 |
| -44,-54,-8 | -1.948 | 0.005999982 | Left inferior temporal gyrus, BA 37 |
| -18,-68,-28 | -1.948 | 0.000999987 | Left cerebellum, crus I, BA 19 |
| -20,-62,-12 | -1.948 | 0.000999987 | Left lingual gyrus, BA 19 |
| -46,-50,-14 | -1.948 | 0.001999974 | Left inferior temporal gyrus, BA 20 |
| 42,-58,-28 | -1.948 | 0.000999987 | Right cerebellum, crus I, BA 37 |
| -46,-62,-14 | -1.948 | 0.003000021 | Left inferior occipital gyrus, BA 37 |
| 8,-58,-38 | -1.947 | 0.032999992 | Cerebellum, vermic lobule IX |
| -24,-74,-16 | -1.946 | 0.000999987 | Left fusiform gyrus, BA 18 |
| -36,-60,-28 | -1.945 | 0.000999987 | Left cerebellum, crus I, BA 37 |
| 8,-68,-30 | -1.945 | 0.001999974 | Right cerebellum, hemispheric lobule VIII |
| 28,-66,-16 | -1.94 | 0.001999974 | Right fusiform gyrus, BA 19 |
| -32,52,10 | -1.938 | 0.01700002 | Left middle frontal gyrus, BA 10 |
| 30,-20,54 | -1.922 | 0.000999987 | (undefined), BA 4 |
| 14,-78,34 | -1.922 | 0.000999987 | Right cuneus cortex, BA 19 |
| 0,-68,34 | -1.922 | 0.000999987 | Left precuneus |
| 4,-68,38 | -1.922 | 0.000999987 | Right precuneus, BA 7 |
| -28,-54,52 | -1.922 | 0.032000005 | Left inferior parietal (excluding supramarginal and angular) gyri, BA 7 |
| -6,-70,38 | -1.922 | 0.000999987 | Left precuneus, BA 7 |
| -26,-60,56 | -1.922 | 0.032000005 | Left superior parietal gyrus, BA 7 |
| 24,-64,56 | -1.921 | 0.015999973 | Right superior parietal gyrus, BA 7 |
| 4,-14,56 | -1.92 | 0.003000021 | Right supplementary motor area, BA 6 |
| 30,42,-14 | -1.919 | 0.010999978 | Right middle frontal gyrus, orbital part, BA 11 |
| 0,-62,-30 | -1.918 | 0.001999974 | Cerebellum, vermic lobule VIII |
| -14,-40,-14 | -1.918 | 0.018000007 | Left cerebellum, hemispheric lobule IV / V |
| 38,58,-4 | -1.912 | 0.01700002 | Right middle frontal gyrus, orbital part, BA 10 |
| 24,-24,54 | -1.908 | 0.001999974 | Right hand superior U tract |
| 18,-74,44 | -1.903 | 0.003000021 | Right precuneus, BA 19 |
| 34,56,-6 | -1.902 | 0.018000007 | Right middle frontal gyrus, orbital part, BA 47 |
| 46,10,-26 | -1.901 | 0.010999978 | Right temporal pole, superior temporal gyrus, BA 20 |
| 28,-72,44 | -1.9 | 0.003000021 | Right superior occipital gyrus, BA 7 |
| -54,-54,32 | -1.893 | 0.010999978 | Left angular gyrus, BA 39 |
| -18,24,46 | -1.891 | 0.033999979 | Left frontal superior longitudinal |
| 12,-88,-14 | -1.877 | 0.005999982 | Right lingual gyrus, BA 18 |
| 40,-54,40 | -1.868 | 0.010999978 | Right inferior parietal (excluding supramarginal and angular) gyri, BA 40 |
| -6,-12,56 | -1.865 | 0.003000021 | Left supplementary motor area, BA 6 |
| 26,-40,-28 | -1.853 | 0.010999978 | Right cerebellum, hemispheric lobule IV / V, BA 37 |
| -38,-52,-28 | -1.835 | 0.005999982 | Left cerebellum, hemispheric lobule VI, BA 37 |
| 34,-48,60 | -1.834 | 0.018000007 | Right superior parietal gyrus, BA 2 |
| 48,-20,50 | -1.814 | 0.003000021 | Right postcentral gyrus, BA 4 |
| 32,-52,54 | -1.809 | 0.015999973 | Right inferior parietal (excluding supramarginal and angular) gyri, BA 7 |
| -4,-38,60 | -1.798 | 0.018000007 | Left precuneus, BA 5 |
| 42,50,-6 | -1.79 | 0.018000007 | Right middle frontal gyrus, orbital part, BA 46 |
| 6,24,46 | -1.785 | 0.005999982 | Right supplementary motor area, BA 8 |
| -52,-54,26 | -1.773 | 0.010999978 | Left angular gyrus, BA 22 |
| 50,-32,54 | -1.705 | 0.035000026 | Right postcentral gyrus, BA 2 |
| -52,-44,-8 | -1.694 | 0.035000026 | Left arcuate network, long segment |

**Table S3. Heterogeneity statistics for the areas reported for the meta-analysis of brain activation to emotion in FEP patients versus healthy controls.**

| Coordinates | Region | SDM-Z | Estimate I^2^ | Estimate Q^2^ | Estimate H^2^ |
| --- | --- | --- | --- | --- | --- |
| -38,2,-10 | Left insula, BA 48 | -4.655 | 0 | 0.77148 | 1.00000 |
| -26,-2,-14 | Left amygdala, BA 34 | -4.027 | 0 | 0.90438 | 1.00000 |
| 28,-38,4 | Right hippocampus, BA 37 | -3.95 | 0 | 1.35795 | 1.00000 |
| -18,-10,-12 | Left hippocampus | -4.093 | 0 | 1.38875 | 1.00000 |
| 0,40,4 | Left anterior cingulate / paracingulate gyri | -3.611 | 0 | 1.23011 | 1.00000 |
| 10,46,2 | Right anterior cingulate / paracingulate gyri, BA 10 | -3.184 | 0 | 1.32822 | 1.00000 |
| -42,-72,14 | Left middle occipital gyrus, BA 37 | -3.565 | 0 | 1.26232 | 1.00000 |


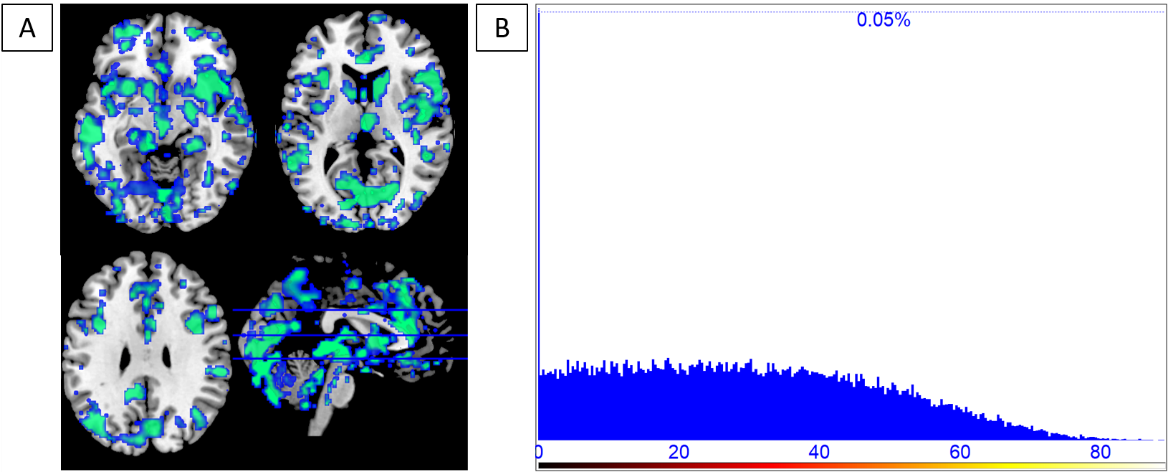
**Figure S1.** **The I^2^ heterogeneity statistic distribution from the meta-analysis of brain activation to emotion in FEP patients versus healthy controls.**

**(A) Whole-brain distribution of the I^2^ statistic. I^2^ increases from blue to cyan. Transparent areas have an I^2^ value of 0. (B) The distribution of the I^2^ statistic in the meta-analysis.**

**Combined meta-analysis of brain activation to emotion in FEP and CHRp compared to healthy controls.**

A combined meta-analysis of t-maps of brain activation to emotional versus neutral stimuli in FEP patients and individuals at CHRp compared to healthy controls showed significantly decreased activation in a large widespread cluster (Z = -(4.253-1.406), k = 77,082, p_TFCE_ < 0.05). The cluster comprised peaks in several brain regions classically involved in emotion processing, such as the left insula (x = -38, y = -4, z = -12, Z = -3.932, p_TFCE_ = 0.000999987), left amygdala (x = -22, y = 2, z = -18, Z = -2.769, p_TFCE_ = 0.000999987), right amygdala (x = 22, y = -2, -12, Z = -3.275, p_TFCE_ = 0.000999987), right hippocampus (x = 32, y = -18, z = -16, Z = -3.523, p_TFCE_ = 0.000999987), left hippocampus (x = -20, y = -8, z = -12, Z = -4.031, p_TFCE_ = 0.000999987), anterior cingulate (x = -4, y = 38, z = 4, Z = -2.974, p_TFCE_ = 0.000999987), and occipital cortex (x = -28, y = -82, z = 18, Z = -3.542, p_TFCE_ = 0.000999987) (Fig. S2).


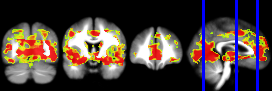
Figure S2. Results of a combined meta-analysis of group-comparison t-maps in both FEP and CHRp populations compared to healthy controls. Results shown on a standard template at p_TFCE_<0.001 for display purposes. Decreasing Z value displayed with increasing warm colours (green, yellow, red).

**References**

1. Modinos G et al. Neural correlates of aberrant emotional salience predict psychotic symptoms and global functioning in high-risk and first-episode psychosis. Soc Cogn Affect Neurosci. 2015;10(10):1429–36. https://doi.org/10.1093/scan/nsv035

2. Tseng HH et al. Corticolimbic dysfunction during facial and prosodic emotional recognition in first-episode psychosis patients and individuals at ultra-high risk. NeuroImage Clin. 2016;12:645–54. https://doi.org/10.1016/j.nicl.2016.09.006

3. Reske M et al. Differential brain activation during facial emotion discrimination in first-episode schizophrenia. J Psychiatr Res. 2009;43(6):592–9. https://doi.org/10.1016/j.jpsychires.2008.10.012

4. Yang C et al. Different levels of facial expression recognition in patients with firstepisode schizophrenia: A functional MRI study. Gen Psychiatry. 2018;31(2):e000014. https://doi.org/10.1136/gpsych-2018-000014

5. Das P et al. Functional disconnections in the direct and indirect amygdala pathways for fear processing in schizophrenia. Schizophr Res. 2007;90(1–3):284–94. https://doi.org/10.1016/j.schres.2006.11.023

6. Hempel A et al. Impairment in basal limbic function in schizophrenia during affect recognition. Psychiatry Res - Neuroimaging. 2003;122(2):115–24. https://doi.org/10.1016/s0925-4927(02)00126-9

7. Villalta-Gil V et al. Functional similarity of facial emotion processing between people with a first episode of psychosis and healthy subjects. Schizophr Res. 2013;149(1–3):35–41. https://doi.org/10.1016/j.schres.2013.06.020

8. Bergé D et al. Limbic activity in antipsychotic naïve first-episode psychotic subjects during facial emotion discrimination. Eur Arch Psychiatry Clin Neurosci. 2014;264(4):271–83. https://doi.org/10.1007/s00406-013-0465-5

9. Knolle F et al. Brain responses to different types of salience in antipsychotic naïve first episode psychosis: An fMRI study. Transl Psychiatry. 2018;8(1):196. https://doi.org/10.1038/s41398-018-0250-3

10. Ebisch SJH et al. Out of touch with reality? Social perception in first-episode schizophrenia. Soc Cogn Affect Neurosci. 2013;8(4):394–403. https://doi.org/10.1093/scan/nss012

11. Catalucci A et al. Neuronal basis of haedonic appraisal in early onset schizophrenia: fMRI investigation. Neuroradiol J. 2011;24(2):264–70. https://doi.org/10.1177/197140091102400216

12. Seiferth NY et al. Increased neural response related to neutral faces in individuals at risk for psychosis. Neuroimage. 2008;40(1):289–97. https://doi.org/10.1016/j.neuroimage.2007.11.020

13. Gee DG et al. Altered age-related trajectories of amygdala-prefrontal circuitry in adolescents at clinical high risk for psychosis: A preliminary study. Schizophr Res. 2012;134(1):1–9. https://doi.org/10.1016/j.schres.2011.10.005

14. Derntl B et al. Empathy in individuals clinically at risk for psychosis: Brain and behaviour. Br J Psychiatry. 2015;207(5):407–13. https://doi.org/10.1192/bjp.bp.114.159004

15. Quarmley M et al. Reduced safety processing during aversive social conditioning in psychosis and clinical risk. Neuropsychopharmacology. 2019;44(13):2247–53. https://doi.org/10.1038/s41386-019-0421-9

16. Lang PJ et al. International affective picture system (IAPS): Technical manual and affective ratings. NIMH Center for the Study of Emotion and Attention. Gainsville, FLNIMH Center for the study of emotion and attention; 1997.

17. Van Der Velde J et al. Lower prefrontal activation during emotion regulation in subjects at ultrahigh risk for psychosis: An fMRI-study. npj Schizophr. 2015;1:15026. https://doi.org/10.1038/npjschz.2015.26
